# Herpesvirus gB: A Finely Tuned Fusion Machine

**DOI:** 10.3390/v7122957

**Published:** 2015-12-11

**Authors:** Rebecca S. Cooper, Ekaterina E. Heldwein

**Affiliations:** Department of Molecular Biology and Microbiology, Tufts University School of Medicine, Boston, MA 02111, USA; rebecca.cooper@tufts.edu

**Keywords:** herpesviruses, cell entry, membrane fusion, glycoproteins, virions, X-ray crystallography, electron microscopy, electron tomography

## Abstract

Enveloped viruses employ a class of proteins known as fusogens to orchestrate the merger of their surrounding envelope and a target cell membrane. Most fusogens accomplish this task alone, by binding cellular receptors and subsequently catalyzing the membrane fusion process. Surprisingly, in herpesviruses, these functions are distributed among multiple proteins: the conserved fusogen gB, the conserved gH/gL heterodimer of poorly defined function, and various non-conserved receptor-binding proteins. We summarize what is currently known about gB from two closely related herpesviruses, HSV-1 and HSV-2, with emphasis on the structure of the largely uncharted membrane interacting regions of this fusogen. We propose that the unusual mechanism of herpesvirus fusion could be linked to the unique architecture of gB.

## 1. Introduction

All viruses must enter living cells to replicate, which requires them to overcome the barrier of the cellular membrane. Enveloped viruses—those coated with a lipid bilayer called an envelope—solve this problem by facilitating the merger of their envelope with the cellular membrane, during which the viral contents are delivered into the cytosol and infection ensues. This process of membrane fusion is initiated by binding of a virus to an appropriate receptor on the surface of the host cell and is catalyzed by a virus-encoded membrane fusion protein, or fusogen. In the majority of enveloped viruses, the receptor-binding and the fusogenic functions are performed by a single protein [1].

Herpesviruses are enveloped double-stranded DNA viruses that establish lifelong latent infections from which they periodically reactivate, primarily causing mucocutaneous lesions. More rarely, they can also cause severe conditions such as encephalitis, keratitis, cancer, and infections in newborns. Herpesvirus entry is a complex process in which fusion alone requires three conserved proteins at minimum—gB, gH, and gL. Fusion also requires additional non-conserved glycoproteins specific to individual herpesviruses [2], and could be further modulated by viral and host proteins not discussed here [3,4,5]. What do all these proteins do and what drives this unusual division of labor? This review addresses these fundamental questions by focusing on the conserved fusogen gB, its structure, function, and distinctions from other viral fusogens. We discuss the complex regulation of gB conformation and activity by gH/gL as well as its own membrane proximal region, transmembrane domain, and CTD. Ultimately, we attempt to link the unusual mechanism of HSV-1 fusion to the unique architecture of gB, the main player in this intricate process.

## 2. Viral Entry and Membrane Fusion

### 2.1. General Mechanism of Viral Fusion

**Figure 1:**
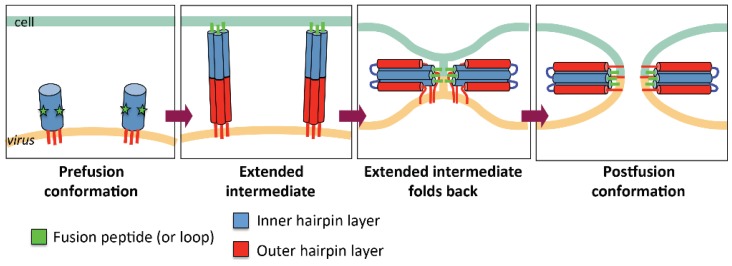
A diagram illustrating viral membrane fusion. Stages in the refolding of a fusogen are indicated. The regions of the fusogen that become the inner and outer layers of the postfusion hairpin are shown in blue and red, respectively. The fusion peptides (or loops) are shown in green.

Viral fusogens mediate the merger of the viral lipid envelope and the cell membrane. These proteins are displayed on the viral surface and anchored into the envelope. In the course of undergoing a thermodynamically favorable conformational rearrangement, fusogens are thought to provide the energy to clear the kinetic barrier to fusion [1]. Prior to the receipt of an environmental signal—typically acidic pH or binding of a cellular receptor—fusogens reside in a metastable prefusion state on the viral membrane (Figure 1). Upon triggering, they expose their previously shielded fusion segments (N-terminal peptides or hydrophobic internal loops) and extend them towards the opposing host cell membrane. This elongated conformation is unstable and rapidly folds back upon itself such that the C-terminal transmembrane anchor of each fusogen protomer, embedded in the viral envelope, is brought into proximity with its host membrane-interacting fusion segment. Formation of this low-energy, trimeric hairpin structure, termed postfusion, therefore draws these opposing membranes together and facilitates their merger.

### 2.2. Three Classes of Viral Fusogens

**Figure 2:**
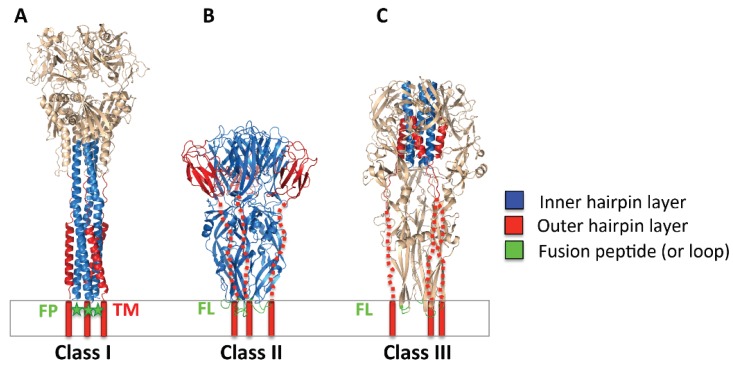
Postfusion structures of representative viral fusogens from each of the three known classes. Class I, PIV3 F (PDB ID 1ZTM); class II, Dengue E (PDB ID 1OK8), class III, VSV G (PDB ID 2CMZ). The inner and outer layers of the postfusion hairpin are shown in blue and red, respectively. Fusion loops (FL) of Dengue E and VSV G are shown in green. The fusion peptide (FP) of PIV3 F, missing from the structure, is indicated with a green star. The extended C-terminal segments in Dengue E and VSV G, unresolved in the structures, are shown as dashed red lines. The TM segments are represented by red cylinders. The membrane is shown schematically. Figure was made with Pymol.

All known viral fusogens belong to one of three different classes (Figure 2) [1] although fusogens of pestiviruses and Hepatitis C may belong to a novel class [6]. Class I is exemplified by Influenza hemagglutinin and includes fusogens from diverse viral families including retroviruses, paramyxoviruses, filoviruses, and coronaviruses, among others. The unifying structural features of class I fusogens are triple-helical prefusion cores, which refold into a six-helix bundle in the postfusion state, and proteolytically generated N-terminal fusion peptides capable of perturbing membranes [7] (Figure 2A) (note, however, that Ebola gp uses a loop [8]). Class II fusogens are found in flaviviruses, e.g., West Nile virus, and have three-domain, beta-sheet-rich structures containing internal fusion loops [9] (Figure 2B). They can be arranged as homodimers or heterodimers in prefusion form and are maintained in this metastable condition through association with a chaperone protein [10,11,12]. Class III fusogens possess elements from both class I and II, containing the former group’s helical core and the internal fusion loops of the latter (Figure 2C). Class III includes glycoprotein G from rhabdoviruses, e.g., Vesicular Stomatitis virus (VSV), gB from herpesviruses, and gp64 from baculoviruses of insects [13]. It is the least well characterized fusogen group, as the structures of both the prefusion and the postfusion forms are known only for VSV G [14,15] and the refolding pathway has not yet been mapped out.

Despite the lack of sequence similarity and large differences in structure, fusion in all three groups is initiated almost exclusively by two common triggers. Binding to a cellular receptor triggers some members of class I, e.g., SARS spike protein [16,17], while exposure to low pH activates Influenza HA (class I) [18], all class II fusogens [10], and the class III fusogens gp64 and VSV G [14,19]. In this later scheme, the protonation of key histidine and/or acidic residues is thought to act as a molecular switch to drive refolding [14,20,21,22]. The triggering mechanisms of some viruses are more complex, requiring multiple steps or cellular interactions; binding to a receptor CD4 and a co-receptor CXCR4 or CCR5 in the case of HIV [23], binding to a receptor followed by low pH exposure for Avian Leukosis Virus [24], or cathepsin cleavage after cellular uptake followed by binding of NPC1, an intracellular cholesterol transporter, for Ebola [25,26]. Salient features of most viral fusion schemes are that the fusogen interacts directly with the triggering stimulus and acts independently, executing the membrane merger without activation by other viral glycoproteins [1]. Paramyxoviruses, in which the class I fusogen F is triggered by interaction with a separate receptor-binding viral protein (HN, H, or G, depending on the paramyxovirus) [27], and herpesviruses, discussed below, represent major departures from this norm.

### 2.3. The Orchestration of Herpesvirus Membrane Fusion

**Figure 3:**
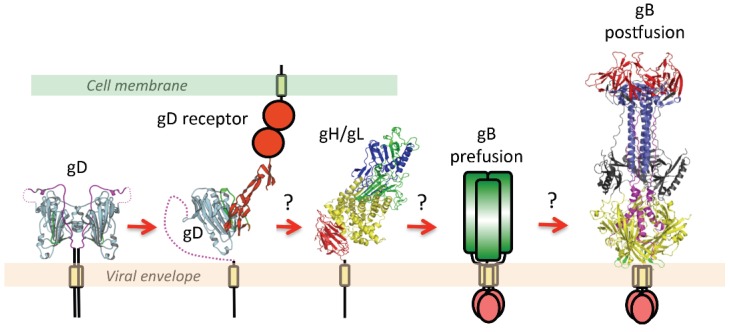
A schematic diagram of essential steps in HSV glycoprotein-mediated fusion. Crystal structures of apo gD (PDB ID 2C36), gD/HVEM complex (PDB ID 1JMA), gH/gL (PDB ID 3M1C), and the postfusion form of gB (PDB ID 2GUM) are shown. The prefusion form of gB has not yet been characterized and is shown schematically. Conformational changes in gD upon receptor binding are well documented. The order of subsequent steps has been proposed but not yet confirmed. Figure was made with Pymol.

In all eight herpesviruses, gB requires the assistance of the conserved heterodimeric gH/gL complex to effect fusion of a virion to its target membrane [2]. This merger can occur at the endosomal or plasma membrane, depending on the identity of the herpesvirus and its target cell. In herpes simplex viruses, HSV-1 and HSV-2, a non-conserved receptor-binding protein, gD, is needed in addition to gH/gL and gB (Figure 3).

According to the current model [28], fusion is initiated when gD binds one of its cellular receptors, nectin-1, a cell adhesion molecule, herpesvirus entry mediator HVEM, a member of the TNFalpha family [29], or modified heparan sulfate [30]. This engagement releases gD from its autoinhibited dimeric state [31,32] and enables it to somehow transmit a signal to gH/gL [33,34,35], which, in turn, activates gB [36,37,38] (Figure 3). This sequential activation process is triggered differently in beta- and gammaherpesviruses. The Epstein-Barr virus (EBV), a gammaherpesvirus, employs glycoprotein gp42, a gD analog, to engage HLA receptor on the surface of B cells [39,40] but uses gH/gL to bind αvβ5, αvβ6, and αvβ8 integrins on epithelial cells directly [41,42]. These integrins also have roles in HSV entry, regulating its route and timing [3], but their involvement in fusion itself is not firmly established. Entry of cytomegalovirus (CMV) into fibroblasts depends on the presence of the trimeric gH/gL/gO complex [43,44] whereas entry into both epithelial and endothelial cells requires a pentameric complex formed by gH/gL, UL128, UL130, and UL131 [45,46] although gH/gL/gO may be important for CMV entry into these latter cell types as well [47].

Regardless of the variation in how their fusogenic machinery is initially triggered, herpesvirus fusion mechanisms converge at the point of gB activation by gH/gL. This process is unusually elaborate in comparison to the fusion schemes of most viruses, which employ a single, directly activated fusogen. Most importantly, in all herpesviruses, gB does not orchestrate fusion on its own and requires the conserved gH/gL plus additional non-conserved partner glycoproteins. Although HSV-1 gB binds the cellular receptor heparan sulfate [48], this interaction only aids in viral attachment and does not initiate fusion. Furthermore, as evidenced by the existence of pH-independent endocytosis and plasma membrane HSV entry routes, gB mediated fusion is not triggered by low pH. Limited pH-induced structural changes do occur in gB, but they do not trigger its global rearrangement [49]. These differences hint that the forces that determine the conformation of gB and the timing of its refolding are unique even among class III fusogens. They also raise numerous questions about how gB interacts with fusion partners and the surrounding lipid bilayer. For example, is the fusogenic refolding of gB restrained or promoted by its interactions with these other proteins? Or, what features of gB, a protein that by comparison to other class III fusogens appears to contain the full complement of machinery needed to effect fusion, make it dependent upon other glycoproteins? Finally, how does the viral membrane in which gB resides shape its structure and activity? A complete structure of gB will accelerate the investigation of these specific questions about its function and begin to unravel many of the broader mysteries of HSV fusion.

## 3. The gB Ectodomain

As noted above, many aspects of gB function are both unique and incompletely understood. Much of what we do know about gB, from its identification as a class III fusogen to details like the position of its fusion loops, derives from the crystal structure of postfusion ectodomain. The major movements executed by gB during fusion can be inferred from this structure, but the accuracy and detail of this reconstruction are limited by the dearth of information on its metastable prefusion conformation. This long-sought structure would also provide invaluable clues as to the mechanism and timing of gB triggering.

### 3.1. Structure of the Postfusion gB Ectodomain

**Figure 4:**
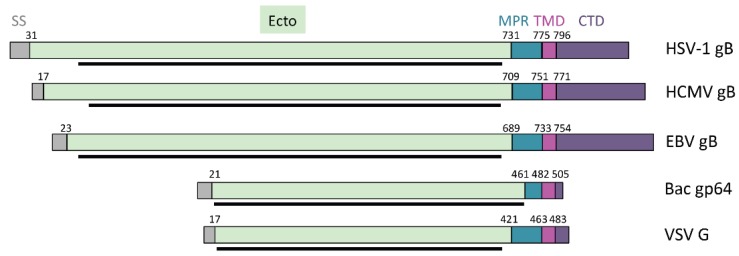
Bar diagram showing the relative lengths of different protein segments in class III fusogens. Signal sequences (SS, grey), ectodomains (ecto, palegreen), membrane proximal regions (MPR, teal), transmembrane domains (TMD, magenta), and cytoplasmic domain (CTD, purple). The black lines below each bar indicate portions of the ectodomains resolved in the crystal structures of each fusogen.

**Figure 5:**
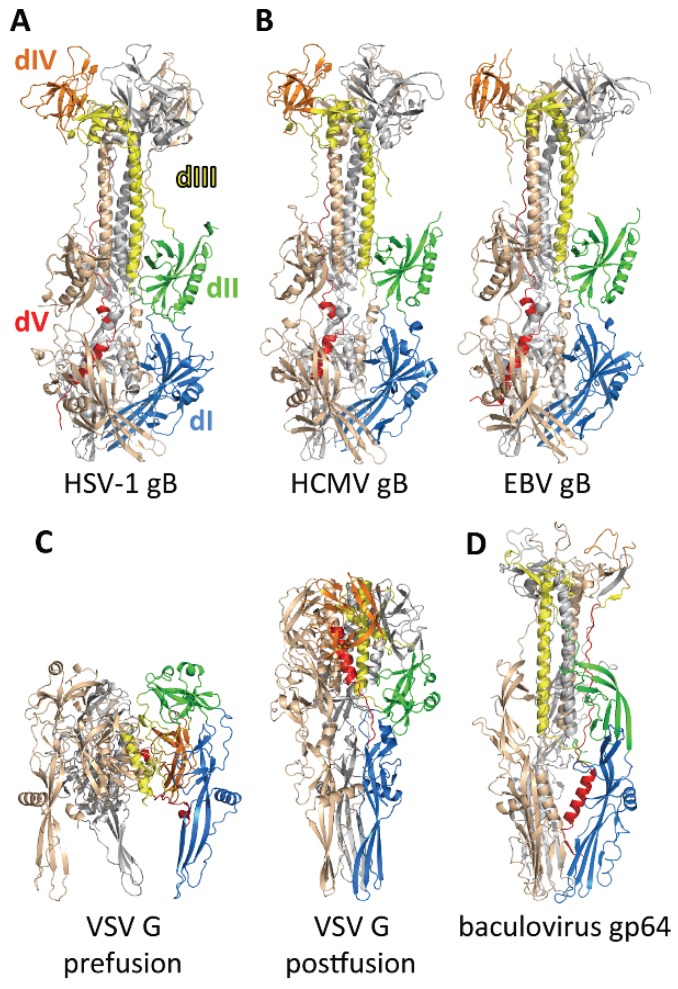
The crystal structures of class III viral fusogens. HSV-1 gB (PDB ID 2GUM), CMV gB (PDB ID 5CXF), EBV gB (PDB ID 3FVC), and baculovirus gp64 (PDB ID 3DUZ) are shown in the postfusion conformations. VSV G is shown in both prefusion and postfusion conformations (PDB ID 2J6J and 2CMZ). A single protomer is each structure is colored by domain: dI, blue; dII, green; dIII, yellow; dIV, orange, and dV, red. The other two protomers are shown in gray and wheat, for ease of comparison. Figure was made with Pymol.

Like all viral fusogens, gB is composed of a large extraviral or ectodomain, which is the conformationally labile “business” end of the molecule, a hydrophobic membrane-proximal region located above the membrane, a single-span helical transmembrane anchor, and an intraviral or cytoplasmic domain (Figure 4). The crystal structure of the HSV-1 gB ectodomain in its postfusion form revealed a trimeric spike in which each protomer is composed of 5 distinct domains arranged into a hairpin shape (Figure 5A) [50]. Domains I–IV of each protomer twist around the equivalent regions of their counterparts, resulting in multiple inter-subunit contacts that contribute to trimer stability. The structure is further reinforced by domain V, which extends from one end of the molecule to the other and packs into a groove between the other two protomers thereby forming the outer layer of the hairpin. A similar overall architecture was observed in the subsequently determined crystal structures of gB ectodomain from EBV [51] and CMV [52,53] (Figure 5B) although individual domain orientations differ between the gB homologs and may reflect virus-specific adaptations.

The hairpin architecture of the gB ectodomain is common to the postfusion structures of viral fusogens from all classes, and since it is especially similar to postfusion VSV G (Figure 5C), identifies gB as a class III fusogen. Individual domains within gB, VSV G, and baculovirus gp64 share a strong resemblance and their spatial arrangements are similar (Figure 5). However, the sequences of these proteins lack any similarity and differ in length by nearly 400 amino acids. Moreover, in marked contrast to HSV gB, conformational changes in VSV G and gp64 are pH-driven and reversible. Taken together, these differences emphasize that not all findings from VSV G are applicable to gB. Nevertheless, knowledge of VSV G will remain a valuable platform for understanding gB-mediated fusion until a cohesive model describing the conformational changes within gB and its interactions with other viral components is developed.

### 3.2. The Prefusion Form of gB

In contrast to our detailed knowledge of the postfusion form of the gB ectodomain, the prefusion form of gB remains uncharacterized, with even its oligomeric state unknown. Whereas all characterized fusogens are trimers in their postfusion forms, their prefusion forms span a range of oligomeric states, from trimers to homodimers or even heterodimers. Thus, the oligomeric state of the prefusion form of gB cannot be deduced from the oligomeric state of other fusogens. Several computational models for prefusion gB have been generated using homology modeling and the structure of the prefusion VSV G as a template [51,54,55]. But, given the significant structural and mechanistic differences between gB and VSV G discussed above, the veracity of such models is unclear.

Experimental characterization of the prefusion form of gB has been hindered in part by a lack of antibodies specific to this conformation. All known neutralizing antibodies to HSV gB, some of which should theoretically bind only the prefusion form and prevent its refolding, also bind the postfusion form [56,57]. Similar antibody reactivity has been observed with the fusogen F of respiratory syncytial virus (RSV), in which two major neutralizing epitopes remain intact and exposed in the postfusion form despite extensive refolding [58,59,60].

The prefusion form of gB has not yet been captured in a form suitable for structural analysis. When the isolated gB ectodomain is expressed, it directly adopts its postfusion conformation [61], a testament to the high stability of this form. Similar behavior has been observed with recombinant ectodomains of several other viral fusogens, such as IAV HA and PIV F, [62,63]. Yet, whereas the prefusion forms of several fusogens have been stabilized by adding C-terminal trimerization tags [64,65,66], gB has so far eluded capture through this approach [61]. Attempts to produce the prefusion form of the gB ectodomain by introducing helix breaking or bulky residues into the postfusion coiled-coil core have likewise failed [61]. These results raise the possibility that the isolated ectodomain of gB lacks an innate ability to adopt its prefusion conformation and that gH/gL, other regions of gB, or perhaps even the membrane, are required to maintain gB in this metastable state *in vivo*.

## 4. Regulatory Regions of gB

While the fusogenic ability of gB derives from its ectodomain, the adjoining membrane proximal region (MPR), transmembrane domain (TMD), and cytoplasmic domain (CTD) are thought to play key roles in its regulation. These membrane interacting regions make up 20% of the protein, a noteworthy fraction in comparison to other fusogens (Figure 4), and are discussed below specifically for HSV. In their regulatory capacity, they may also be indispensable for maintaining gB in its active, prefusion form.

### 4.1. Membrane-Proximal Region

Between the ectodomain and the TMD lies a stretch of 43 amino acids termed the membrane-proximal region (MPR) (Figure 4). Approximately 40% of the residues within the MPR are conserved among alphaherpesviruses, and some of these are invariant among all herpesviruses. Mutation of this highly conserved subset in HSV-1 gB yields viruses with negligible infectivity [67]. The MPR is predicted to be helical and, due to its highly hydrophobic character, probably interacts with the membrane. It may form a pedestal beneath the ectodomain, similarly to the stem-loop region in flavivirus E [68]. In E, a class II fusogen, the MPR folds separately from the ectodomain in the prefusion form but becomes an integral part of the postfusion structure, forming the outer layers of the trimeric hairpin [69]. Since the gB MPR is not a part of the postfusion hairpin [50], it may participate in fusion differently from other MPRs.

One of the main functions of the MPR may be to shield the fusion loops prior to gB triggering [70,71]. The fusion loops of class III fusogens are expected to be near the viral membrane in both pre- and postfusion structures [14,15], and they readily interact with membranes when exposed on the surface of recombinant HSV-1 ectodomains [72]. In contrast, ectodomain-MPR constructs do not [70,71]. As few as nine N-terminal MPR residues (731 to 739) are sufficient to prevent liposome binding, and two phenylalanines (F732 and F738) are essential for this blocking activity [71]. The MPR therefore appears to isolate the fusion loops until they can be deployed for the merger of the viral and target cell membranes. However, one caveat to this conclusion is that, because membrane-binding experiments were carried out with the postfusion gB ectodomain, it is unclear to what extent it applies to the prefusion form.

While the N-terminal portion of the MPR could shield the fusion loops to prevent their unproductive insertion into the viral membrane, this domain may also contribute directly to the fusion process by facilitating lipid mixing. This idea is bolstered by the proximity of the MPR to the membrane, its highly hydrophobic composition, and studies of other class III fusogens. In baculovirus gp64, a trio of leucines in the MPR (referred to as pre-transmembrane domain) assists with the formation of the fusion pore [73]. The C-terminal MPR segment of VSV G, rich in aromatic residues, is also required for cell-cell fusion [74]. Moreover, a VSV G fragment that contains only the C-terminal 14-amino-acids of its MPR, the TMD and the cytotail, is sufficient to induce membrane deformation, initiate hemifusion, and reduce the overall energy barrier to fusion [75]. Similar direct findings on the role of the gB MPR are lacking. Many mutants with insertions and deletions in this area are poorly expressed on the cell surface, possibly due to gB misfolding [71,76,77,78]. Nonetheless, the fusogenicity of those that reach the membrane is very low, demonstrating that the MPR is essential for fusion.

### 4.2. Transmembrane Domain

**Figure 6:**
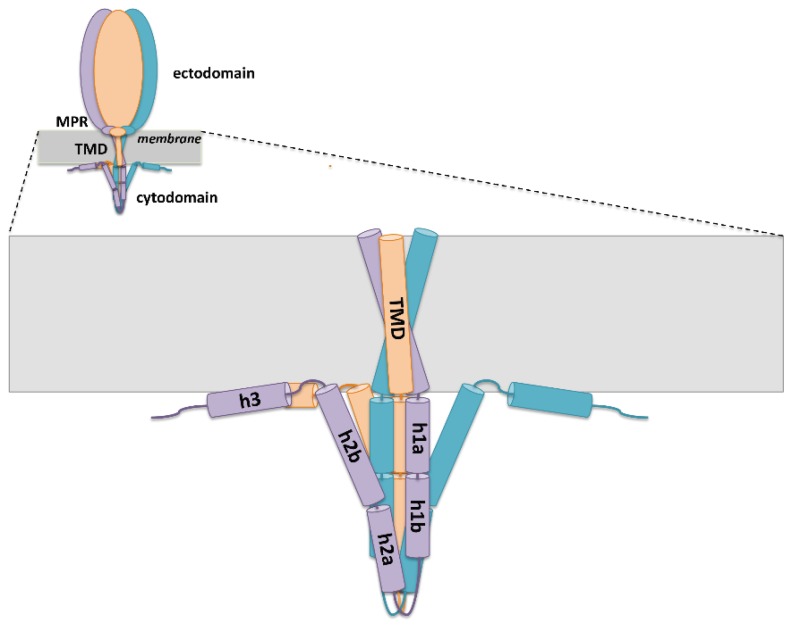
A model of the HSV-1 gB. Both the entire protein and a close-up view of the TMD and the CTD are shown. The ectodomain and the MPR regions are represented by ovals of different sizes. TMD helices are shown in a funnel configuration, one possible way in which they may be arranged. The CTD has a proteolytically resistant core and is predicted to contain three helices: h1, h2, and h3. These may be intact or interrupted by short linkers (as shown). Helices h2 and h3 are thought to interact with the membrane.

Between the MPR and CTD of gB lies a single-pass transmembrane domain (TMD) (Figure 4 and Figure 6) containing a predicted 20–22 residues [77,79], This segment is likely alpha-helical, as this is the most energetically favorable arrangement for a hydrophobic span of this length [80,81]. Fusion is arrested at the hemifusion stage when the HSV-1 gB TMD is replaced with a lipid anchor [82], similarly to what has been observed in other fusogens, e.g., VSV G and Influenza HA [83,84]. Thus, the gB TMD is more than a mere membrane anchor and plays an essential role in the later stages of fusion.

The TMDs of other fusogens have been proposed to contribute to fusion by enlarging the fusion pore [85] or acting as a conduit to transfer lipids between the opposing membranes [86]. Analagous findings on the gB TMD are lacking, but insights about its architecture and function can be drawn from other fusogens and membrane-spanning helices in general. The length of the gB TMD is similar to several other fusogens [87,88,89] and slightly exceeds the minimal 20 residues theoretically needed to span an average 30-Å lipid bilayer hydrophobic core [80,90]. It is therefore tempting to speculate that gB must traverse the membrane via nearly the shortest path, parallel to the membrane normal. However, the bilayer thickness varies considerably with its composition [90] and, because the average TMD of crystallized proteins is both longer and more tilted than sequence-based predictions suggest [80,91], this arrangement is far from certain. Furthermore, the TMDs of some fusogens can be shortened to 16 or 17 aa before fusion is abolished due to poor surface expression [87] or, more interestingly, impaired completion of the fusion pore [92]. These studies emphasize that fusogen TMDs must span the membrane, but suggest there is some latitude in the position of full-length TMDs. This ability to adopt multiple transmembrane orientations, depending on the stage of fusion, has been proposed to be integral to the mechanism of paramyxovirus F, which has an unusually long 25+ residue TMD [86]. Although the gB TMD is of a more typical length, a similar principle may shape its fusogenic mechanism.

Like its size, the TMD composition could provide clues to how it may interact with and shape the membrane around it. Alphaherpesvirus gB TMDs contain six conserved alanines, five conserved leucines, and three conserved glycines, an amino acid that is overrepresented in fusogen transmembrane helices [89]. Glycines destabilize helices by reducing side chain interactions [93] and glycine-induced helix kinks could accommodate large transmembrane domain rearrangements [94], a strong possibility during fusion. Conversely, amino acids like leucine form efficient contacts with the side chains of multiple residues on the same helix face (*i* to *i + 3* and *i + 4*) and thus enhance helix rigidity [95]. The activity of many fusogens depends on the overall structural and biochemical properties of their TMDs [87,88,96], so other properties of the gB TMD may derive from groups of amino acids. There are several conserved glycine-alanine pairs in the TMD that are separated by three residues, enabling them to form GxxxG-like motifs in which their absent or small side chains align to form a void [97]. The impact of GxxxG-like motifs on the structure of helical domains is likely similar to, if less extreme than, the extensively studied canonical GxxxG motif [98]. This sequence is present in the TMDs of both HIV-1 gp41 [88] and VSV G [89]. It increases helix backbone dynamics in synthetic VSV G TMD peptides and may yield highly mobile TMD helices in full-length VSV G that could interact strongly with the aliphatic chains of membrane lipids and cause lipid splaying [99], wherein the two lipid tails are pulled apart during fusion to facilitate lipid bilayer mixing. Indeed, replacement of the VSV G glycines additively impairs fusion pore formation, with complete inactivity resulting from the loss of both glycines [89]. Transmembrane helices containing the GxxxG sequence also display strong homotypic binding tendencies [100] that may facilitate their association in fusogens [101]. Consistent with this prediction, the cryoEM structure of HIV-1 gp41 shows its three TMD helices forming a left-handed coiled-coil with a 35° crossing angle [102]. In gB, misfolding observed upon the addition of a heterologous trimerization domain to the CTD N-terminus suggests that the TMD helices are separated as they exit the cytoplasmic face of the membrane [103]. However, the extracellular arrangement of these helices is unknown and, in the absence of direct structural information on the TMD, it is impossible to assess whether GxxxG-like motifs cause these helices to cross or otherwise associate. Since the gB TMD is an active participant in the membrane fusion process [82], additional studies are warranted to elucidate its mechanistic contributions to fusion and tie them to its form and composition.

### 4.3. Cytoplasmic Domain

The strong similarity of the gB ectodomain to other class III fusogens suggests that they share a common fusion machinery blueprint. Yet, striking differences emerge in comparing their intraviral, or cytoplasmic, domains (CTDs). While the CTDs of VSV G and gp64 are unstructured and short, at only 29 and 7 residues respectively, the 109-residue long HSV-1 gB CTD forms a trimer that is predicted to be approximately 50% alpha-helical [103] (Figure 6). This extensive secondary structure develops only in the presence of anionic liposomes, detergent micelles or mixed micelles, with which it interacts strongly, and an isolated CTD is only 25% alpha-helical in solution. This dramatic increase in folding is accompanied by the expansion of its proteolytically resistant core, which strongly indicates that the organization and function of this unique domain depend upon its association with the membrane [103].

The gB CTD appears to negatively regulate fusion. Although HSV infection typically does not cause cell-cell fusion, clinical isolates of HSV-1 with truncations, point mutations, or insertions within the gB CTD form multinucleated cells termed syncytia [104,105,106,107]. Similar hyperfusogenic phenotypes have been observed for gB CTD truncations in other herpesviruses (Varicella-Zoster virus, Epstein-Barr virus, pseudorabies virus) [108,109,110]. It has been proposed that syncytial truncations in HSV-1 gB act by disrupting membrane binding by the CTD and, consequently, preclude the formation of important secondary structure elements [111]. Likewise, a 14-residue C-terminal truncation in EBV gB produced a hyperfusogenic protein that could mediate fusion at lower temperatures, more rapidly, and in response to weaker signals from gH/gL than WT EBV gB [112]. No changes in the oligomerization of gB or the strength of its interaction with gH/gL were found, and the authors attribute the apparent lower activation energy of the shortened mutant to reduced interaction of its CTD with the membrane.

Although the gB CTD may need to bind the membrane to restrain fusion, this is not the sole determinant of its regulatory ability. Several syncytial CTD point mutants, both naturally occurring and engineered, display wild-type or stronger membrane interactions [103]. Instead, a variety of local structural changes are observed at the mutation sites. The CTD may act as a clamp that braces against the virion envelope to prevent fusion, and altering this inhibitory conformation could effectively uncouple the ectodomain from its restraining influence. This theory that is supported by the finding that HSV-1 gB with hyperfusogenic point mutations appears to have a lowered fusion energy barrier and is hypersensitive to signals from gH/gL [113]. Perhaps fusion is affected by inside-out-signaling, in which the CTD “status” is conveyed through the TMD and to the ectodomain [114,115]. Point mutations, though seemingly minor, could change the transmission of such a message through the disruption of intramolecular contacts or secondary structure elements.

Among class III fusogens, the role of the CTD as a clamp may be unique to herpesviruses, an unsurprising fact considering the small size of the VSV G and gp64 cytotails (Figure 4). Indeed, a HCMV “gB-G” construct in which the CTD, transmembrane and a portion of the membrane proximal region of gB were replaced with the VSV G transmembrane spans and cytotail could drive membrane fusion [116]. Yet, examples of fusion inhibition by the CTD are also found in Class I fusogens. Some paramyxovirus PIV5 F protein variants possess extended cytotails that reduce normal syncytia formation through trimerization [114]. Proteolytic removal of 16 C-terminal residues is a prerequisite for fusogenic conformational rearrangements in the murine leukemia virus Env protein [117]. Finally, despite having little effect on membrane fusion, truncation of the HIV-1 Env cytotail greatly changes the antigenic properties of its ectodomain suggesting that the genuinely native prefusion form requires the intact cytotail [118]. Nonetheless, the gB regulatory mechanism is uniquely dependent upon a large, structured CTD that interacts with the membrane.

It is clear that HSV-1 fusion is a tightly regulated process in which the CTD of gB is of critical importance. Controlling fusion is in essence preventing the premature conversion of metastable prefusion gB to postfusion gB, a function that the CTD achieves by interacting with the viral envelope and, probably, with the TMD. This restraint is only effective if the integrity of the CTD is preserved, and aberrant fusion occurs in response to slight perturbations of its structure or disruption of CTD-membrane contacts. Common approaches to the study of membrane proteins, such as isolation of the ectodomain or solubilization of full-length protein, inherently perturb these critical interactions, and may explain the elusiveness of prefusion gB. Obtaining the prefusion gB structure will require new tactics that can isolate full-length gB in a more native-like environment.

## 5. Role of gH/gL Interactions

According to the current model, fusion in herpesviruses requires a signal passed from the gH/gL heterodimer to gB [37]. The HSV-2 gH/gL heterodimer, which is very similar in sequence to HSV-1, has a large boot-shaped ectodomain composed of three gH domains and the entirety of the gL scaffold protein [38]. The most N-terminal gH domain is stabilized by folding around gL, and the C-terminal domain extends into a single transmembrane pass and 14-amino-acid cytotail. How gH/gL regulates gB is unknown, but it has been found that their C termini come into proximity only in the presence of activated gD [36]. This observation suggests that gB and gH/gL do not constitutively associate, and thus that gH/gL is likely to activate, rather than repress, gB fusogenic activity.

### 5.1. The gH/gL Ectodomain

gH exhibits a spectrum of conservation along its sequence that appears integral to its communication with gB. Extensive variability is seen in the membrane distal H1 domains of gH/gL heterodimers from different viruses, and it may enable them to receive a variety of activating signals. The highly conserved central H2 and membrane adjacent H3 domains then translate these diverse inputs into a common message for transmission to gB. Given that gB itself is the most highly conserved herpesvirus glycoprotein, tolerating few changes to preserve its fusogenic capability, gH/gL has been proposed as an adaptor needed for it to function in many different systems [119].

A stable complex involving gH/gL and gB has never been captured, but preliminary information on how and where their ectodomains might interact has been obtained through investigation of two strongly neutralizing gH/gL antibodies. The first, LP11, binds in a location that blocks gB–gH/gL association, suggesting its that its epitope overlaps the contact area for these glycoproteins [38]. Another antibody, 52S, binds opposite the LP11 site and allows gH/gL binding to gB. The attachment of 52S may prevent binding of activated gD [34].

### 5.2. The gH Cytotail

Regulation of gB by the gH/gL heterodimer is typically attributed to its ectodomain, but the short gH cytotail may be involved in this process as well. Although HSV-1 gH/gL has been reported to activate gB when expressed in a soluble form or from the opposing membrane, it does so far less efficiently than as a full-length construct in the same membrane as gB [33]. Indeed, attaching the HSV-1 gH ectodomain to the membrane with a GPI lipid anchor renders it inactive [82], and replacement of the gH transmembrane domain and cytotail with the corresponding domains of other proteins produced chimeras that could not mediate cell-cell fusion when coexpressed with the other requisite HSV-1 glycoproteins [120].

The ability of the gH cytotail to regulate fusion has been examined in several mutational studies. Truncation of the 14-amino-acid HSV-1 gH cytotail by more than 6 residues impairs syncytium formation [121]. This effect has been attributed to the destruction of the serine-valine-proline (SVP) motif of residues 830–832 [122], and fusion events mediated by both WT gB and A855V hyperfusogenic gB are further reduced in the presence of gH SAP mutants [123], where a V831A substitution is introduced into gH SVP (gH truncated after P832). However, there are other indications that the length of the gH cytotail is a more critical determinant of gH activity than its sequence. A systematic study of HSV-1 gH cytotail truncation mutants demonstrated that shortening of the gH cytotail progressively curtailed cell-cell fusion [113], and a similar conclusion has been reached regarding VZV gH cytotail, the action of which was entirely contingent upon length rather than sequence [124]. Furthermore, despite the prediction that the HSV-1 gH cytotail is unstructured [125], insertions are capable of retarding or abrogating both cell-cell fusion and viral infectivity [126].

In lieu of stably binding the gB CTD, the gH cytotail has been proposed to exert its influence via inside-out signaling [123]. However, gH cytotail truncations do not alter the ectodomain conformation [113,120,126]. In light of the clear trend toward reduced fusion with reduced cytotail length, a more likely possibility is that this domain promotes fusion through a transient, steric mechanism. It may act as a wedge to split tightly associated protomers of the gB CTD clamp, freeing the ectodomain to undergo fusogenic refolding [113].

It is interesting to contrast the effect shortening the HSV-1 gH cytotail with similar mutations to the VZV cytotail. Shortening of the VZV gH cytotail increases fusion, indicating that gH restrains fusion instead of promoting it [124]. Although slightly different (nonequivalent) mutations were studied in each case, these opposing results may relate to the contrasting activity of gB in these proteins. Syncytia formation is a constitutive part of VZV pathogenesis that requires attenuation—excessive cell-cell fusion is associated with low infectivity. In contrast, syncytia are not seen in WT HSV-1 fusion, which is already tightly controlled by the built in safety-latch of gBcyto and requires prompting. Thus, the function of gH may be dictated by the “set-point” of the gB fusogen it accompanies.

## 6. Conclusions

Crossing the threshold of its intended host cell’s membrane is a key challenge that a virus must accomplish to establish an infection. In enveloped viruses like HSV-1 and HSV-2, this entry process entails the merger of the virion envelope with the endosomal or plasma membrane and requires the coordination of many proteins. Ultimately, though, it is always the membrane fusogen gB that does the work of joining these membranes.

As with other viral fusogens, much information about the function of gB has been gleaned by studying the stable, postfusion conformation of its ectodomain. This domain contains the primary hardware needed for fusion—fusion loops to grab to target membrane and deployable, spring-loaded arms to pull it into the viral membrane. Most fusogens are able to work independently because this machinery is directly activated upon binding to cellular receptors or the sensing of pH changes. However, gB activation in HSV-1 and HSV-2 requires the participation of the both the gH/gL heterodimer and the gD receptor binding protein. Furthermore, gB mediated fusion is restrained by its substantial CTD, which lacks an equivalent among the short cytotails of other Class III fusogens.

Although the mechanism by which the CTD controls fusion is unknown, its function is sensitive to minor structural changes and is contingent upon contact with the viral envelope. In conjunction with the other membrane interacting regions of gB, the membrane proximal region and transmembrane domain, it may act to stabilize the metastable prefusion conformation. If so, these three smaller domains essentially modulate the conformation of the ectodomain and the timing of fusion. It is perhaps no surprise that the ectodomain has only been isolated in the postfusion form. Once fusion is initiated, the MPR and TMD may also facilitate lipid mixing and formation of the fusion pore. Finally, the requirement to release a CTD clamp could also explain why, despite possessing ectodomain fusion machinery that is highly similar to that of other independently acting class III fusogens, gB is reliant on other proteins. In essence, the unique activation scheme of herpesviruses is tied to the unusual structure of its fusogen, gB.

## References

[1] Harrison S.C. (2015). Viral membrane fusion. Virology.

[2] Heldwein E.E., Krummenacher C. (2008). Entry of herpesviruses into mammalian cells. Cell. Mol. Life Sci..

[3] Gianni T., Salvioli S., Chesnokova L.S., Hutt-Fletcher L.M., Campadelli-Fiume G. (2013). Alphavbeta6- and alphavbeta8-integrins serve as interchangeable receptors for HSV gH/gL to promote endocytosis and activation of membrane fusion. PLoS Pathog..

[4] Gianni T., Massaro R., Campadelli-Fiume G. (2015). Dissociation of HSV gL from gH by alphavbeta6- or alphavbeta8-integrin promotes gH activation and virus entry. Proc. Natl Acad Sci. USA.

[5] Satoh T., Arii J., Suenaga T., Wang J., Kogure A., Uehori J., Arase N., Shiratori I., Tanaka S., Kawaguchi Y. (2008). PILRalpha is a herpes simplex virus-1 entry coreceptor that associates with glycoprotein B. Cell.

[6] Li Y., Modis Y. (2014). A novel membrane fusion protein family in Flaviviridae?. Trends Microbiol..

[7] Lamb R.A., Jardetzky T.S. (2007). Structural basis of viral invasion: Lessons from paramyxovirus F. Curr. Opin. Struct. Biol..

[8] Lee J.E., Fusco M.L., Hessell A.J., Oswald W.B., Burton D.R., Saphire E.O. (2008). Structure of the Ebola virus glycoprotein bound to an antibody from a human survivor. Nature.

[9] Modis Y. (2013). Class II fusion proteins. Adv. Exp. Med. Biol..

[10] Kielian M. (2006). Class II virus membrane fusion proteins. Virology.

[11] Li L., Lok S.M., Yu I.M., Zhang Y., Kuhn R.J., Chen J., Rossmann M.G. (2008). The flavivirus precursor membrane-envelope protein complex: Structure and maturation. Science.

[12] Voss J.E., Vaney M.C., Duquerroy S., Vonrhein C., Girard-Blanc C., Crublet E., Thompson A., Bricogne G., Rey F.A. (2010). Glycoprotein organization of Chikungunya virus particles revealed by X-ray crystallography. Nature.

[13] Backovic M., Jardetzky T.S. (2009). Class III viral membrane fusion proteins. Curr. Opin. Struct. Biol..

[14] Roche S., Rey F.A., Gaudin Y., Bressanelli S. (2007). Structure of the prefusion form of the vesicular stomatitis virus glycoprotein G. Science.

[15] Roche S., Bressanelli S., Rey F.A., Gaudin Y. (2006). Crystal structure of the low-pH form of the vesicular stomatitis virus glycoprotein G. Science.

[16] Zelus B.D., Schickli J.H., Blau D.M., Weiss S.R., Holmes K.V. (2003). Conformational changes in the spike glycoprotein of murine coronavirus are induced at 37 degrees C either by soluble murine CEACAM1 receptors or by pH 8. J. Virol..

[17] Matsuyama S., Taguchi F. (2009). Two-step conformational changes in a coronavirus envelope glycoprotein mediated by receptor binding and proteolysis. J. Virol..

[18] Bullough P.A., Hughson F.M., Skehel J.J., Wiley D.C. (1994). Structure of influenza haemagglutinin at the pH of membrane fusion. Nature.

[19] Kadlec J., Loureiro S., Abrescia N.G., Stuart D.I., Jones I.M. (2008). The postfusion structure of baculovirus gp64 supports a unified view of viral fusion machines. Nat. Struct. Mol. Biol..

[20] Mair C.M., Meyer T., Schneider K., Huang Q., Veit M., Herrmann A. (2014). A histidine residue of the influenza virus hemagglutinin controls the pH dependence of the conformational change mediating membrane fusion. J. Virol..

[21] Kampmann T., Mueller D.S., Mark A.E., Young P.R., Kobe B. (2006). The role of histidine residues in low-pH-mediated viral membrane fusion. Structure.

[22] Roche S., Albertini A.A., Lepault J., Bressanelli S., Gaudin Y. (2008). Structures of vesicular stomatitis virus glycoprotein: Membrane fusion revisited. Cell. Mol. Life Sci..

[23] Wilen C.B., Tilton J.C., Doms R.W. (2012). Molecular mechanisms of HIV entry. Adv. Exp. Med. Biol..

[24] Mothes W., Boerger A.L., Narayan S., Cunningham J.M., Young J.A. (2000). Retroviral entry mediated by receptor priming and low pH triggering of an envelope glycoprotein. Cell.

[25] Chandran K., Sullivan N.J., Felbor U., Whelan S.P., Cunningham J.M. (2005). Endosomal proteolysis of the Ebola virus glycoprotein is necessary for infection. Science.

[26] Cote M., Misasi J., Ren T., Bruchez A., Lee K., Filone C.M., Hensley L., Li Q., Ory D., Chandran K. (2011). Small molecule inhibitors reveal Niemann-Pick C1 is essential for Ebola virus infection. Nature.

[27] Lamb R.A., Paterson R.G., Jardetzky T.S. (2006). Paramyxovirus membrane fusion: Lessons from the F and HN atomic structures. Virology.

[28] Eisenberg R.J., Atanasiu D., Cairns T.M., Gallagher J.R., Krummenacher C., Cohen G.H. (2012). Herpes virus fusion and entry: A story with many characters. Viruses.

[29] Spear P.G. (2004). Herpes simplex virus: Receptors and ligands for cell entry. Cell Microbiol..

[30] Tiwari V., O’Donnell C., Copeland R.J., Scarlett T., Liu J., Shukla D. (2007). Soluble 3-O-sulfated heparan sulfate can trigger herpes simplex virus type 1 entry into resistant Chinese hamster ovary (CHO-K1) cells. J. Gen. Virol..

[31] Lazear E., Carfi A., Whitbeck J.C., Cairns T.M., Krummenacher C., Cohen G.H., Eisenberg R.J. (2008). Engineered disulfide bonds in herpes simplex virus type 1 gD separate receptor binding from fusion initiation and viral entry. J. Virol..

[32] Krummenacher C., Supekar V.M., Whitbeck J.C., Lazear E., Connolly S.A., Eisenberg R.J., Cohen G.H., Wiley D.C., Carfi A. (2005). Structure of unliganded HSV gD reveals a mechanism for receptor-mediated activation of virus entry. EMBO J..

[33] Atanasiu D., Saw W.T., Cohen G.H., Eisenberg R.J. (2010). Cascade of events governing cell-cell fusion induced by herpes simplex virus glycoproteins gD, gH/gL, and gB. J. Virol..

[34] Atanasiu D., Cairns T.M., Whitbeck J.C., Saw W.T., Rao S., Eisenberg R.J., Cohen G.H. (2013). Regulation of herpes simplex virus gB-induced cell-cell fusion by mutant forms of gH/gL in the absence of gD and cellular receptors. MBio.

[35] Gianni T., Amasio M., Campadelli-Fiume G. (2009). Herpes simplex virus gD forms distinct complexes with fusion executors gB and gH/gL in part through the C-terminal profusion domain. J. Biol. Chem.

[36] Atanasiu D., Whitbeck J.C., Cairns T.M., Reilly B., Cohen G.H., Eisenberg R.J. (2007). Bimolecular complementation reveals that glycoproteins gB and gH/gL of herpes simplex virus interact with each other during cell fusion. Proc. Natl. Acad. Sci. USA.

[37] Atanasiu D., Whitbeck J.C., de Leon M.P., Lou H., Hannah B.P., Cohen G.H., Eisenberg R.J. (2010). Bimolecular complementation defines functional regions of herpes simplex virus gB that are involved with gH/gL as a necessary step leading to cell fusion. J. Virol..

[38] Chowdary T.K., Cairns T.M., Atanasiu D., Cohen G.H., Eisenberg R.J., Heldwein E.E. (2010). Crystal structure of the conserved herpesvirus fusion regulator complex gH-gL. Nat. Struct. Mol. Biol..

[39] Li Q., Spriggs M.K., Kovats S., Turk S.M., Comeau M.R., Nepom B., Hutt-Fletcher L.M. (1997). Epstein-Barr virus uses HLA class II as a cofactor for infection of B lymphocytes. J. Virol..

[40] Mullen M.M., Haan K.M., Longnecker R., Jardetzky T.S. (2002). Structure of the Epstein-Barr virus gp42 protein bound to the MHC class II receptor HLA-DR1. Mol. Cell.

[41] Chesnokova L.S., Hutt-Fletcher L.M. (2011). Fusion of Epstein-Barr virus with epithelial cells can be triggered by αvβ5 in addition to αvβ6 and αvβ8, and integrin binding triggers a conformational change in glycoproteins gHgL. J. Virol..

[42] Chesnokova L.S., Nishimura S.L., Hutt-Fletcher L.M. (2009). Fusion of epithelial cells by Epstein-Barr virus proteins is triggered by binding of viral glycoproteins gHgL to integrins αvβ6 or αvβ8. Proc. Natl. Acad. Sci. USA.

[43] Vanarsdall A.L., Chase M.C., Johnson D.C. (2011). Human cytomegalovirus glycoprotein gO complexes with gH/gL, promoting interference with viral entry into human fibroblasts but not entry into epithelial cells. J. Virol..

[44] Wille P.T., Knoche A.J., Nelson J.A., Jarvis M.A., Johnson D.C. (2010). A human cytomegalovirus gO-null mutant fails to incorporate gH/gL into the virion envelope and is unable to enter fibroblasts and epithelial and endothelial cells. J. Virol..

[45] Wang D., Shenk T. (2005). Human cytomegalovirus virion protein complex required for epithelial and endothelial cell tropism. Proc. Natl. Acad. Sci. USA.

[46] Ryckman B.J., Jarvis M.A., Drummond D.D., Nelson J.A., Johnson D.C. (2006). Human cytomegalovirus entry into epithelial and endothelial cells depends on genes UL128 to UL150 and occurs by endocytosis and low-pH fusion. J. Virol..

[47] Zhou M., Lanchy J.M., Ryckman B.J. (2015). Human cytomegalovirus gH/gL/gO promotes the fusion step of entry into all cell types, whereas gH/gL/UL128–131 broadens virus tropism through a distinct mechanism. J. Virol..

[48] Laquerre S., Argnani R., Anderson D.B., Zucchini S., Manservigi R., Glorioso J.C. (1998). Heparan sulfate proteoglycan binding by herpes simplex virus type 1 glycoproteins B and C, which differ in their contributions to virus attachment, penetration, and cell-to-cell spread. J. Virol..

[49] Stampfer S.D., Lou H., Cohen G.H., Eisenberg R.J., Heldwein E.E. (2010). Structural basis of local, pH-dependent conformational changes in glycoprotein B from herpes simplex virus type 1. J. Virol..

[50] Heldwein E.E., Lou H., Bender F.C., Cohen G.H., Eisenberg R.J., Harrison S.C. (2006). Crystal structure of glycoprotein B from herpes simplex virus 1. Science.

[51] Backovic M., Longnecker R., Jardetzky T.S. (2009). Structure of a trimeric variant of the Epstein-Barr virus glycoprotein B. Proc. Natl. Acad. Sci. USA.

[52] Chandramouli S., Ciferri C., Nikitin P.A., Calo S., Gerrein R., Balabanis K., Monroe J., Hebner C., Lilja A.E., Settembre E.C. (2015). Structure of HCMV glycoprotein B in the postfusion conformation bound to a neutralizing human antibody. Nat. Commun..

[53] Burke H.G., Heldwein E.E. (2015). Crystal structure of the human cytomegalovirus glycoprotein B. PLoS Pathog..

[54] Atanasiu D., Saw W.T., Gallagher J.R., Hannah B.P., Matsuda Z., Whitbeck J.C., Cohen G.H., Eisenberg R.J. (2013). Dual split protein-based fusion assay reveals that mutations to herpes simplex virus (HSV) glycoprotein gB alter the kinetics of cell-cell fusion induced by HSV entry glycoproteins. J. Virol..

[55] Gallagher J.R., Atanasiu D., Saw W.T., Paradisgarten M.J., Whitbeck J.C., Eisenberg R.J., Cohen G.H. (2014). Functional fluorescent protein insertions in herpes simplex virus gB report on gB conformation before and after execution of membrane fusion. PLoS Pathog..

[56] Bender F.C., Samanta M., Heldwein E.E., de Leon M.P., Bilman E., Lou H., Whitbeck J.C., Eisenberg R.J., Cohen G.H. (2007). Antigenic and mutational analyses of herpes simplex virus glycoprotein B reveal four functional regions. J. Virol..

[57] Cairns T.M., Fontana J., Huang Z.Y., Whitbeck J.C., Atanasiu D., Rao S., Shelly S.S., Lou H., Ponce de Leon M., Steven A.C. (2014). Mechanism of neutralization of herpes simplex virus by antibodies directed at the fusion domain of glycoprotein B. J. Virol..

[58] McLellan J.S., Chen M., Leung S., Graepel K.W., Du X., Yang Y., Zhou T., Baxa U., Yasuda E., Beaumont T. (2013). Structure of RSV fusion glycoprotein trimer bound to a prefusion-specific neutralizing antibody. Science.

[59] McLellan J.S., Yang Y., Graham B.S., Kwong P.D. (2011). Structure of respiratory syncytial virus fusion glycoprotein in the postfusion conformation reveals preservation of neutralizing epitopes. J. Virol..

[60] McLellan J.S. (2015). Neutralizing epitopes on the respiratory syncytial virus fusion glycoprotein. Curr. Opin. Virol..

[61] Vitu E., Sharma S., Stampfer S.D., Heldwein E.E. (2013). Extensive mutagenesis of the HSV-1 gB ectodomain reveals remarkable stability of its postfusion form. J. Mol. Biol..

[62] Yin H.S., Paterson R.G., Wen X., Lamb R.A., Jardetzky T.S. (2005). Structure of the uncleaved ectodomain of the paramyxovirus (hPIV3) fusion protein. Proc. Natl. Acad. Sci. USA.

[63] Chen J., Wharton S.A., Weissenhorn W., Calder L.J., Hughson F.M., Skehel J.J., Wiley D.C. (1995). A soluble domain of the membrane-anchoring chain of influenza virus hemagglutinin (HA2) folds in *Escherichia coli* into the low-pH-induced conformation. Proc. Natl. Acad. Sci. USA.

[64] Yin H.S., Wen X., Paterson R.G., Lamb R.A., Jardetzky T.S. (2006). Structure of the parainfluenza virus 5 F protein in its metastable, prefusion conformation. Nature.

[65] Sissoeff L., Mousli M., England P., Tuffereau C. (2005). Stable trimerization of recombinant rabies virus glycoprotein ectodomain is required for interaction with the p75NTR receptor. J. Gen. Virol..

[66] Stevens J., Corper A.L., Basler C.F., Taubenberger J.K., Palese P., Wilson I.A. (2004). Structure of the uncleaved human H1 hemagglutinin from the extinct 1918 influenza virus. Science.

[67] Wanas E., Efler S., Ghosh K., Ghosh H.P. (1999). Mutations in the conserved carboxy-terminal hydrophobic region of glycoprotein gB affect infectivity of herpes simplex virus. J. Gen. Virol..

[68] Zhang X., Ge P., Yu X., Brannan J.M., Bi G., Zhang Q., Schein S., Zhou Z.H. (2013). Cryo-EM structure of the mature dengue virus at 3.5-A resolution. Nat. Struct. Mol. Biol..

[69] Modis Y., Ogata S., Clements D., Harrison S.C. (2004). Structure of the dengue virus envelope protein after membrane fusion. Nature.

[70] Maurer U.E., Zeev-Ben-Mordehai T., Pandurangan A.P., Cairns T.M., Hannah B.P., Whitbeck J.C., Eisenberg R.J., Cohen G.H., Topf M., Huiskonen J.T. (2013). The structure of herpesvirus fusion glycoprotein B-bilayer complex reveals the protein-membrane and lateral protein-protein interaction. Structure.

[71] Shelly S.S., Cairns T.M., Whitbeck J.C., Lou H., Krummenacher C., Cohen G.H., Eisenberg R.J. (2012). The membrane-proximal region (MPR) of herpes simplex virus gB regulates association of the fusion loops with lipid membranes. MBio.

[72] Hannah B.P., Cairns T.M., Bender F.C., Whitbeck J.C., Lou H., Eisenberg R.J., Cohen G.H. (2009). Herpes simplex virus glycoprotein B associates with target membranes via its fusion loops. J. Virol..

[73] Li Z., Blissard G.W. (2009). The pre-transmembrane domain of the Autographa californica multicapsid nucleopolyhedrovirus GP64 protein is critical for membrane fusion and virus infectivity. J. Virol..

[74] Jeetendra E., Ghosh K., Odell D., Li J., Ghosh H.P., Whitt M.A. (2003). The membrane-proximal region of vesicular stomatitis virus glycoprotein G ectodomain is critical for fusion and virus infectivity. J. Virol..

[75] Jeetendra E., Robison C.S., Albritton L.M., Whitt M.A. (2002). The membrane-proximal domain of vesicular stomatitis virus G protein functions as a membrane fusion potentiator and can induce hemifusion. J. Virol..

[76] Zheng Z., Maidji E., Tugizov S., Pereira L. (1996). Mutations in the carboxyl-terminal hydrophobic sequence of human cytomegalovirus glycoprotein B alter transport and protein chaperone binding. J. Virol..

[77] Rasile L., Ghosh K., Raviprakash K., Ghosh H.P. (1993). Effects of deletions in the carboxy-terminal hydrophobic region of herpes simplex virus glycoprotein gB on intracellular transport and membrane anchoring. J. Virol..

[78] Lin E., Spear P.G. (2007). Random linker-insertion mutagenesis to identify functional domains of herpes simplex virus type 1 glycoprotein B. Proc. Natl. Acad. Sci. USA.

[79] Gilbert R., Ghosh K., Rasile L., Ghosh H.P. (1994). Membrane anchoring domain of herpes simplex virus glycoprotein gB is sufficient for nuclear envelope localization. J. Virol..

[80] Arkin I.T., Brunger A.T. (1998). Statistical analysis of predicted transmembrane α-helices. Biochim. Biophys. Acta.

[81] Engelman D.M., Steitz T.A., Goldman A. (1986). Identifying nonpolar transbilayer helices in amino acid sequences of membrane proteins. Annu. Rev. Biophys. Biophys. Chem..

[82] Jones N.A., Geraghty R.J. (2004). Fusion activity of lipid-anchored envelope glycoproteins of herpes simplex virus type 1. Virology.

[83] Kemble G.W., Danieli T., White J.M. (1994). Lipid-anchored influenza hemagglutinin promotes hemifusion, not complete fusion. Cell.

[84] Odell D., Wanas E., Yan J., Ghosh H.P. (1997). Influence of membrane anchoring and cytoplasmic domains on the fusogenic activity of vesicular stomatitis virus glycoprotein G. J. Virol..

[85] Markosyan R.M., Cohen F.S., Melikyan G.B. (2000). The lipid-anchored ectodomain of influenza virus hemagglutinin (GPI-HA) is capable of inducing nonenlarging fusion pores. Mol. Biol. Cell.

[86] Bissonnette M.L., Donald J.E., DeGrado W.F., Jardetzky T.S., Lamb R.A. (2009). Functional analysis of the transmembrane domain in paramyxovirus F protein-mediated membrane fusion. J. Mol. Biol..

[87] Li Z., Blissard G.W. (2009). The Autographa californica multicapsid nucleopolyhedrovirus GP64 protein: Analysis of transmembrane domain length and sequence requirements. J. Virol..

[88] Miyauchi K., Komano J., Yokomaku Y., Sugiura W., Yamamoto N., Matsuda Z. (2005). Role of the specific amino acid sequence of the membrane-spanning domain of human immunodeficiency virus type 1 in membrane fusion. J. Virol..

[89] Cleverley D.Z., Lenard J. (1998). The transmembrane domain in viral fusion: Essential role for a conserved glycine residue in vesicular stomatitis virus G protein. Proc. Natl. Acad. Sci. USA.

[90] Lee A.G. (2003). Lipid-protein interactions in biological membranes: A structural perspective. Biochim. Biophys. Acta.

[91] Ulmschneider M.B., Sansom M.S. (2001). Amino acid distributions in integral membrane protein structures. Biochim. Biophys. Acta.

[92] Armstrong R.T., Kushnir A.S., White J.M. (2000). The transmembrane domain of influenza hemagglutinin exhibits a stringent length requirement to support the hemifusion to fusion transition. J. Cell Biol..

[93] Pace C.N., Scholtz J.M. (1998). A helix propensity scale based on experimental studies of peptides and proteins. Biophys. J..

[94] Yi M., Cross T.A., Zhou H.X. (2009). Conformational heterogeneity of the M2 proton channel and a structural model for channel activation. Proc. Natl. Acad. Sci. USA.

[95] Quint S., Widmaier S., Minde D., Hornburg D., Langosch D., Scharnagl C. (2010). Residue-specific side-chain packing determines the backbone dynamics of transmembrane model helices. Biophys. J..

[96] Liao M., Kielian M. (2005). The conserved glycine residues in the transmembrane domain of the Semliki Forest virus fusion protein are not required for assembly and fusion. Virology.

[97] Cymer F., Veerappan A., Schneider D. (2012). Transmembrane helix-helix interactions are modulated by the sequence context and by lipid bilayer properties. Biochim. Biophys. Acta.

[98] Senes A., Engel D.E., DeGrado W.F. (2004). Folding of helical membrane proteins: The role of polar, GxxxG-like and proline motifs. Curr. Opin. Struct. Biol..

[99] Stelzer W., Langosch D. (2012). Sequence-dependent backbone dynamics of a viral fusogen transmembrane helix. Protein Sci..

[100] Russ W.P., Engelman D.M. (2000). The GxxxG motif: A framework for transmembrane helix-helix association. J. Mol. Biol..

[101] Unterreitmeier S., Fuchs A., Schaffler T., Heym R.G., Frishman D., Langosch D. (2007). Phenylalanine promotes interaction of transmembrane domains via GxxxG motifs. J. Mol. Biol..

[102] Mao Y., Wang L., Gu C., Herschhorn A., Desormeaux A., Finzi A., Xiang S.H., Sodroski J.G. (2013). Molecular architecture of the uncleaved HIV-1 envelope glycoprotein trimer. Proc. Natl. Acad. Sci. USA.

[103] Silverman J.L., Greene N.G., King D.S., Heldwein E.E. (2012). Membrane requirement for folding of the herpes simplex virus 1 gB cytodomain suggests a unique mechanism of fusion regulation. J. Virol..

[104] Cai W.H., Gu B., Person S. (1988). Role of glycoprotein B of herpes simplex virus type 1 in viral entry and cell fusion. J. Virol..

[105] Baghian A., Huang L., Newman S., Jayachandra S., Kousoulas K.G. (1993). Truncation of the carboxy-terminal 28 amino acids of glycoprotein B specified by herpes simplex virus type 1 mutant amb1511-7 causes extensive cell fusion. J. Virol..

[106] Diakidi-Kosta A., Michailidou G., Kontogounis G., Sivropoulou A., Arsenakis M. (2003). A single amino acid substitution in the cytoplasmic tail of the glycoprotein B of herpes simplex virus 1 affects both syncytium formation and binding to intracellular heparan sulfate. Virus Res..

[107] Gage P.J., Levine M., Glorioso J.C. (1993). Syncytium-inducing mutations localize to two discrete regions within the cytoplasmic domain of herpes simplex virus type 1 glycoprotein B. J. Virol..

[108] Nixdorf R., Klupp B.G., Karger A., Mettenleiter T.C. (2000). Effects of truncation of the carboxy terminus of pseudorabies virus glycoprotein B on infectivity. J. Virol..

[109] Garcia N.J., Chen J., Longnecker R. (2013). Modulation of Epstein-Barr virus glycoprotein B (gB) fusion activity by the gB cytoplasmic tail domain. MBio.

[110] Heineman T.C., Hall S.L. (2002). Role of the varicella-zoster virus gB cytoplasmic domain in gB transport and viral egress. J. Virol..

[111] Chowdary T.K., Heldwein E.E. (2010). Syncytial phenotype of C-terminally truncated herpes simplex virus type 1 gB is associated with diminished membrane interactions. J. Virol..

[112] Chen J., Zhang X., Jardetzky T.S., Longnecker R. (2014). The Epstein-Barr virus (EBV) glycoprotein B cytoplasmic C-terminal tail domain regulates the energy requirement for EBV-induced membrane fusion. J. Virol..

[113] Rogalin H.B., Heldwein E.E. (2015). The interplay between the HSV-1 gB cytodomain and the gH cytotail during cell-cell fusion. J. Virol..

[114] Waning D.L., Russell C.J., Jardetzky T.S., Lamb R.A. (2004). Activation of a paramyxovirus fusion protein is modulated by inside-out signaling from the cytoplasmic tail. Proc. Natl. Acad. Sci. USA.

[115] Aguilar H.C., Anderson W.F., Cannon P.M. (2003). Cytoplasmic tail of Moloney murine leukemia virus envelope protein influences the conformation of the extracellular domain: Implications for mechanism of action of the R Peptide. J. Virol..

[116] Kirchmeier M., Fluckiger A.C., Soare C., Bozic J., Ontsouka B., Ahmed T., Diress A., Pereira L., Schodel F., Plotkin S., Dalba C., Klatzmann D., Anderson D.E. (2014). Enveloped virus-like particle expression of human cytomegalovirus glycoprotein B antigen induces antibodies with potent and broad neutralizing activity. Clin. Vaccine Immunol..

[117] Loving R., Wu S.R., Sjoberg M., Lindqvist B., Garoff H. (2012). Maturation cleavage of the murine leukemia virus Env precursor separates the transmembrane subunits to prime it for receptor triggering. Proc. Natl. Acad. Sci. USA.

[118] Chen J., Kovacs J.M., Peng H., Rits-Volloch S., Lu J., Park D., Zablowsky E., Seaman M.S., Chen B. (2015). Effect of the cytoplasmic domain on antigenic characteristics of HIV-1 envelope glycoprotein. Science.

[119] Fan Q., Longnecker R., Connolly S.A. (2015). A functional interaction between herpes simplex virus 1 glycoprotein gH/gL domains I and II and gD is defined by using alphaherpesvirus gH and gL chimeras. J. Virol..

[120] Harman A., Browne H., Minson T. (2002). The transmembrane domain and cytoplasmic tail of herpes simplex virus type 1 glycoprotein H play a role in membrane fusion. J. Virol..

[121] Wilson D.W., Davis-Poynter N., Minson A.C. (1994). Mutations in the cytoplasmic tail of herpes simplex virus glycoprotein H suppress cell fusion by a syncytial strain. J. Virol..

[122] Browne H.M., Bruun B.C., Minson A.C. (1996). Characterization of herpes simplex virus type 1 recombinants with mutations in the cytoplasmic tail of glycoprotein H. J. Gen. Virol..

[123] Silverman J.L., Heldwein E.E. (2013). Mutations in the cytoplasmic tail of herpes simplex virus 1 gH reduce the fusogenicity of gB in transfected cells. J. Virol..

[124] Yang E., Arvin A.M., Oliver S.L. (2014). The cytoplasmic domain of varicella-zoster virus glycoprotein H regulates syncytia formation and skin pathogenesis. PLoS Pathog..

[125] Kamen D.E., Gross S.T., Girvin M.E., Wilson D.W. (2005). Structural basis for the physiological temperature dependence of the association of VP16 with the cytoplasmic tail of herpes simplex virus glycoprotein H. J. Virol..

[126] Jackson J.O., Lin E., Spear P.G., Longnecker R. (2010). Insertion mutations in herpes simplex virus 1 glycoprotein H reduce cell surface expression, slow the rate of cell fusion, or abrogate functions in cell fusion and viral entry. J. Virol..

